# Robust image quality evaluation in optical coherence tomography of skin using global, region-independent metrics

**DOI:** 10.1038/s41598-026-56302-1

**Published:** 2026-06-08

**Authors:** Elnaz Babaee, Mehdi Boostani, Mostafa Charmi, Kamran Avanaki

**Affiliations:** 1 Richard and Loan Hill Department of Bioengineering, University of Illinois at Chicago, Chicago, IL USA; 2 Department of Electrical Engineering, University of Zanjan, Zanjan, Iran; 3 Department of Dermatology, Venereology and Dermatooncology, Semmelweis University, Budapest, 1085 Hungary

**Keywords:** Optical coherence tomography, OCT, Image quality metrics, Image enhancement evaluation, Computational biology and bioinformatics, Engineering, Mathematics and computing, Optics and photonics

## Abstract

Optical coherence tomography (OCT) is a powerful imaging modality for visualizing tissue microstructures, but its utility is often limited by artifacts such as speckle, intensity decay, and blurring. While numerous image enhancement algorithms have been proposed to address these issues, the field lacks robust, objective metrics to consistently quantify image quality, hindering fair comparison and development of such methods. Existing metrics, such as signal-to-noise ratio (SNR) and contrast-to-noise ratio (CNR), depend heavily on user-selected regions of interest, introducing substantial variability. In this work, we introduce a fully automated framework that segments OCT skin images into air, signal, and noise regions and defines three global image quality metrics: noise-free pixel ratio (NFPR), global SNR (gSNR), and global contrast (gCN). These metrics analyze the entire image without requiring ROI selection, enabling reproducible and user-independent evaluations that correlate with both acquisition parameters and human visual perception. Extensive validation on skin OCT datasets demonstrates that the proposed metrics provide more consistent and stable characterization of image quality compared to conventional ROI-based metrics under the tested conditions. These results suggest that the proposed framework can support objective evaluation of OCT image quality and facilitate the development and benchmarking of image enhancement methods.

## Introduction

**Fig. 1:**
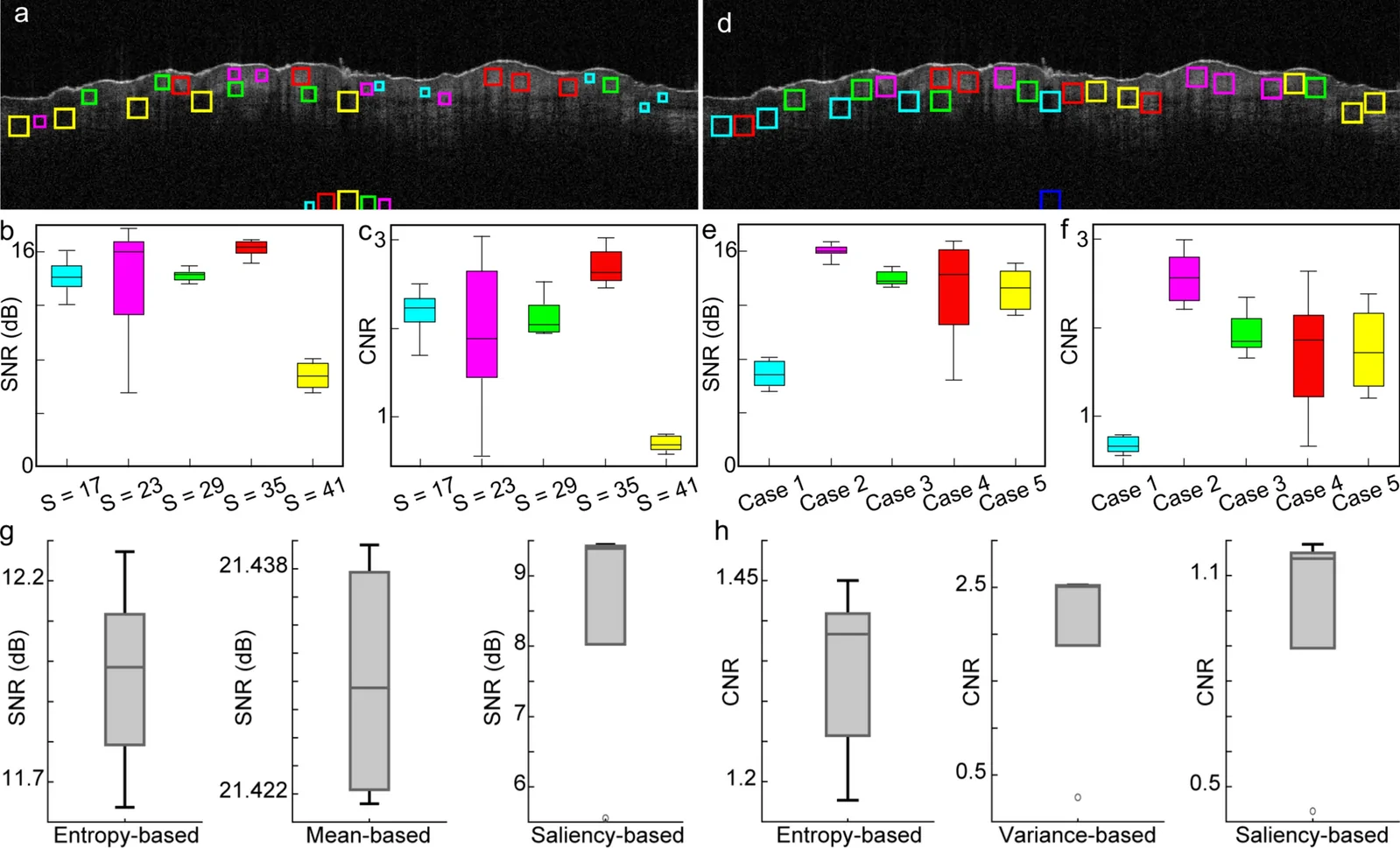
Variation in SNR and CNR values based on the selection of ROIs: (**a**) ROIs with varying sizes selected at different locations. Box plots represent the variation of: (**b**) SNR values, and (**c**) CNR values when ROIs with different sizes are selected. S represents ROI size. (**d**) Same-size ROIs at different locations. Box plots represent the variation of: (**e**) SNR values, and (**f**) CNR values when ROIs with the same size are selected. (**g**, **h**) SNR and CNR values calculated using three adaptive ROI-selection strategies: entropy-based selection of ROIs with the highest information content, statistical optimization based on maximum mean intensity for SNR and minimum variance for CNR, and saliency-based selection of ROIs with the highest structural prominence and edge density.

Optical coherence tomography (OCT) is an imaging modality used to capture 3-dimensional images of tissue micro-structures utilizing low-coherence light. Despite producing high-resolution images, OCT imaging faces challenges in accurately discerning tissue structures within its images, primarily due to the presence of artifacts such as speckle, intensity decay, and blurring^1^. Numerous signal and image processing algorithms have been developed to tackle these artifacts and improve the quality of OCT images^2–15^. Evaluating the performance of these algorithms typically involves using quantitative metrics. Nevertheless, existing quality metrics encounter challenges when applied to OCT images due to their high sensitivity to location. This limitation hinders the fair and accurate comparison between different algorithms. The most commonly used quality metrics for evaluating OCT image quality improvement algorithms are signal-to-noise ratio (SNR) and contrast-to-noise ratio (CNR)^16–25^. SNR compares the signal of an object in the OCT image to its background noise. CNR is a measure of signal fluctuations relative to background noise. SNR is defined as $\mathrm{SNR}=10{\mathrm{log}}_{10}{\mu}_{r}^{2}/{\sigma}_{b}^{2}$, and CNR is defined as $\mathrm{CNR}=\left({\mu}_{r}-{\mu}_{b}\right)/\sqrt{{\sigma}_{r}^{ 2}+{\sigma}_{b}^{ 2}}$ , where μ_*b*_ and σ_*b*_^2^ represent the mean and variance of the background, and μ_r_ and σ_r_^2^ represent the mean and variance of the pixel intensities in the region of interest (ROI)^26^. In other words, SNR specifies how well the morphological features of the imaging target (e.g., tissue) appear on a noisy background (unwanted or random fluctuations of image pixels), and CNR is used to assess the visibility and distinguishability of different structures or features within the image in the presence of noise. Currently, SNR and CNR are usually calculated from the average of multiple arbitrarily preselected small, same-sized regions of an OCT image (signal) and one region in the background (noise). To demonstrate the significant variation of the SNR and CNR values calculated within the same image but based on ROIs chosen from different parts of the of the image, we calculated local SNR and CNR on an OCT image of the skin. The variation in the calculated SNR and CNR values with different sizes of ROI (shown in Fig. 1a), is shown in Fig. 1b and c. Finally, we randomly selected 5 ROIs with the same size of ROI (see Fig. 1d), and using the same noisy ROI, calculated the SNR and CNR values and plotted the observed results (see Fig. 1e and f). In both experiments, a significant variation was observed between the SNR and CNR values calculated within the same image. To eliminate potential bias associated with manual ROI selection and to ensure the reproducibility of the quantitative analysis, a transition was made from a selective sampling approach to an automated, adaptive ROI selection framework. Initially, the primary boundary of the skin surface was identified using an edge-based detection strategy to define the signal-bearing region. A 10-pixel safety margin was then applied below this interface to avoid boundary artifacts. The underlying signal region was subsequently dividedd into overlapping blocks of 25 × 25 pixels, utilizing a single-pixel sliding window shift to maximize spatial sampling density. To objectively identify the most representative signal areas, three distinct adaptive metrics were implemented: (i) an entropy-based method, where five ROIs with the highest information content were selected; (ii) a statistical optimization method, where ROIs with the maximum mean were selected for SNR calculation and those with minimum variance for CNR estimation, and (iii) a saliency-based method, where five ROIs with the highest structural prominence and edge density were identified. For the background, one ROI was selected from the region exhibiting the lowest entropy and mean intensity across all methods. As illustrated in Fig. 1g and h, although the adaptive ROIs converged toward relatively stable spatial locations, significant variations in the resulting SNR and CNR values were observed. These findings demonstrate that even within a standardized automated framework, the choice of the statistical metrics significantly influences the reported SNR and CNR metrics in OCT imaging. Such large variability makes the evaluation of the quality of the image not conclusive: for example, the selection of regions with strong signal heavily skews the result. Uncertainty in how well SNR, CNR, and other abovemenetioned quality metrics assess image quality retards the development of newer and better algorithms. To address this limitation, we propose a fundamentally different framework for OCT image quality assessment based on global, segmentation-driven statistical analysis. Specifically, we introduce three metrics—noise-free pixel ratio (NFPR), global signal-to-noise ratio (gSNR), and global contrast (gCN)—which are computed over the entire image after partitioning OCT data into air, signal, and noise regions using a physics-informed segmentation approach. Unlike conventional ROI-based metrics, the proposed framework is fully automated and eliminates user-dependent variability.

It is important to distinguish this work from existing no-reference image quality assessment (NR-IQA) methods^27–30^ such as naturalness image quality evaluator (NIQE), blind/referenceless image spatial quality evaluator (BRISQUE)^28,31^, perception-based image quality evaluator (PIQE)^32^, and convolutional neural network-based image quality assessment (CNN-IQA)^33^. NR-IQA methods typically rely on learned statistical models derived from natural image statistics or require large training datasets, and their outputs are often not directly interpretable in terms of OCT signal formation. In contrast, the proposed metrics are explicitly grounded in the physical and statistical properties of OCT imaging, including the known behavior of signal attenuation and noise distributions. As such, our approach provides interpretable, acquisition-aware quality measures that are particularly suited for evaluating OCT image formation and system performance. Therefore, rather than replacing NR-IQA approaches, the proposed framework complements them by providing a domain-specific, training-free, and physically interpretable alternative for OCT image quality evaluation.

## Materials and methods

### OCT imaging system

The images analyzed in this study were acquired using a multi-beam, swept-source OCT (SS-OCT) system (Vivosight, Michelson Diagnostic Inc.)^34^. The OCT system has a hand-held scanning probe for skin imaging (central wavelength of 1305 ± 15 nm). The lateral and axial resolutions are 7.5 μm and 10 μm, respectively.

### Dataset

All experimental protocols and imaging procedures involving human participants were reviewed and approved by the Wayne State University Institutional Review Board (IRB). All methods were carried out in accordance with the relevant institutional guidelines and regulations, the approved IRB protocol, and the principles of the Declaration of Helsinki. Written informed consent was obtained from all participants before OCT imaging. Our dataset is composed of 315 stacks of OCT images taken from the forearm, back of hand, forehead, neck, and palm of 63 healthy individuals with different skin types, aged between 20 and 60 years. Each stack has 50 images from the same imaging plane. Each image is 460 × 1500 pixels (1.5 mm × 6 mm). Images were acquired by eight different operators using the OCT system at various times.

### Signal and noise regions detection

#### OCT a-line intensity profile

OCT measures the interference of light returning from the sample with a known reference. After signal acquisition (in wavenumber or time), a Fourier transform is performed to obtain the depth-resolved reflectivity (the “A-line”). When forming an OCT image of the skin, the reflectivity from deeper layers drop below the detector noise level (or the system’s noise floor), so the measured intensities are noise-dominated. In the noise-only regime, the intensity values follow a characteristic statistical distribution.

#### Coefficient of variation profile

*N* pixel intensity values in an OCT A-line *i* $({\mathrm{A}-\mathrm{line}}_{i}\in {\mathrm{R}}^{\mathrm{M}\times 1})$ form a sequence, (${\mathrm{Seq}}_{i}\in {\mathrm{R}}^{\mathrm{N}\times 1})$. The coefficient of variation (CV) is calculated as the ratio of the standard deviation to the mean of the sequence. The CV profile signifies the degree of variation across the image. This process is repeated for each A-line, resulting in a CV map that corresponds to a B-scan OCT image. In noise-dominated regions, the intensity distribution in OCT is often approximated by an exponential model, for which the mean and standard deviation are of similar magnitude. As a result, the coefficient of variation tends to approach values on the order of unity. However, this relationship should be interpreted as an approximate statistical behavior rather than a strict equality, as the exact value may vary depending on system characteristics, reconstruction methods, and noise properties. In contrast, signal-bearing regions typically exhibit lower relative variability, leading to reduced CV values. Therefore, the transition between signal-dominant and noise-dominant regions can be identified through changes in the CV profile, even when the exact threshold varies across datasets. This behavior often appears as a relatively low-CV region followed by a higher-CV region, which motivates the use of CV as a practical feature for automated separation of signal and noise in OCT A-lines.

#### Change points detection method

To identify statistically significant transitions within each OCT A-line, we used the built-in MATLAB function “findabruptchangepts”. For each A-line, the algorithm estimates the change-point locations automatically from the measured intensity or CV profile by partitioning the signal into piecewise segments and minimizing the residual error between the data and the corresponding segment means. An empirical estimate of a mean for each section is computed. Then deviation of the mean from the estimate is measured at each point. The total residual error is calculated for all points in each section. Last, the location of the division point is adjusted to minimize the total residual error. More details can be found in^35,36^.

#### Segmentation algorithm

**Fig. 2:**
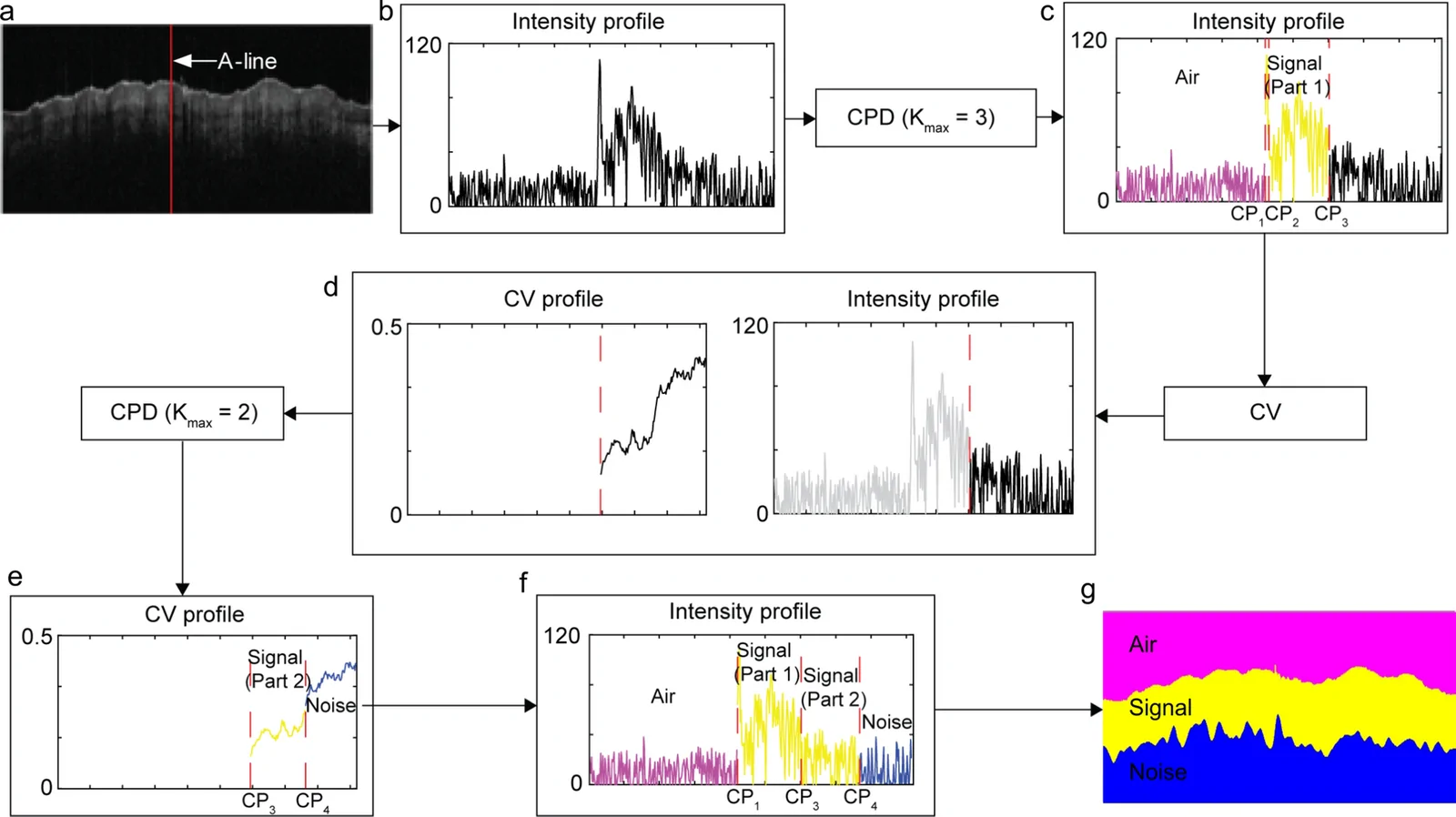
Flowchart of the segmentation algorithm. (**a**) Original B-scan to be analyzed, (**b**) intensity profile of the A-line indicated in (a) by a red line, (**c**) application of the CPD technique to intensity profile identifies 3 change points (CPs) including signal between CP1 and CP3 (signal Part 1), (**d**) intensity profile and CV profile of the segment to the right of CP3, (**e**) CPD technique applied to CV profile identifies one additional CP (CP4) in this segment. We note that the mean CV to the left of CP4 is lower than the mean CV to the right of CP4, and thus we can consider the segment to the left of CP4 to be signal (Part 2), (**f**) signal (yellow) is identified as the region of the A-line between CP1 and CP4, (g) the analysis is completed on all of the A-lines in the B-scan. CPD: changepoints detection, CV: coefficient of variation.

A flowchart of the algorithm to specify the signal and noise regions in the image is depicted in Fig. 2. This algorithm consists of two key stages and leads to the partitioning of each A-line and OCT image into three distinct regions (air, signal, and noise) through the use of the changepoints detection (CPD) technique. The CPD technique identifies abrupt changes (CPs) in the mean value of intensity or CV within an A-line. In general, the CPD technique is applied twice, where the maximum permissible number of CPs (K_max_) is pre-defined each time, the technique is applied. The first time, the entire A-line intensity profile is segmented. The second time, only that part of the A-line that corresponds to the final segment of the intensity profile from the first step is segmented.

Initially, the intensity profile of every A-line in a B-scan is acquired (Fig. 2a). Analysis of the alterations in the intensity profile (Fig. 2b) reveals a distinct pattern: a low-intensity area representing the air region is observed initially (segment 1), followed by a sudden increase in intensity upon reaching the stratum corneum (SC) layer, the outermost layer of the skin, where we see a small region with high intensity (segment 2). As we continue down the A-line, the intensity gradually diminishes, yet a region of relatively high intensity remains detectable (segment 3). However, towards the bottom of the A-line, the intensities decrease significantly, rendering no discernible information available (segment 4). Consequently, our aim is to identify the CPs that distinguish the four segments by considering each A-line individually. To achieve this, we employ the CPD method on the intensity profile and set the maximum number of allowed change points, Kmax, to 3. This value serves only as an upper bound for the CPD search; the actual change-point locations are estimated automatically from each A-line based on the measured intensity profile. The choice of Kmax = 3 is motivated by the expected statistical structure of skin OCT A-lines, which typically include air, an initial signal rise at the tissue interface, a signal-bearing tissue region, and a deeper noise-dominated region. Upon applying this method to every A-line, we discover up to four regions demarcated by red dashed vertical lines on the intensity profile (Fig. 2c). The leftmost region is classified as the air region, while the rightmost region encompasses both noise region and a portion of the signal region. At this point, distinguishing between signal and noise regions solely based on intensity profile is unfeasible due to the low amplitude and minimal disparity in intensities. The region situated in the middle exhibits the most pronounced intensity levels across the A-line and includes the SC layer. This section may consist of either one or two sub-regions, depending upon the disparity in intensity between the pixels of intensity fluctuations. Nevertheless, the entirety of the middle region is regarded as signal (Part 1 in Fig. 2c). The first application of CPD is complete.

In the next phase, the CV profile for each A-line is calculated. We start with the first 25 pixels to calculate the first CV value, which we assign to the 25^th^ pixel and then slide down (1 step) the sequence until reaching the end of the the SC layer and the subsequent pixels, as well as the pattern intensity profile, which results in a CV axial profile corresponding to an A-line. Then, the segment of the CV profile that corresponds to the final region of the intensity profile, consisting of both noise and the remaining part of the signal, is separated out (see Fig. 2d). The CPD method is utilized on the CV profile in this section to establish a boundary between signal and noise regions (Fig. 2e). To identify the transition between residual signal and noise, Kmax was set to 2. This value represents the maximum number of allowable change points, while the actual change-point location is determined automatically from the CV profile of each A-line. This choice was intended to accommodate local variability in the CV profile while preserving the expected signal-to-noise partitioning behavior. The partitioning of the desired segment of the CV profile into a maximum.

### Proposed quality metrics

**Fig. 3:**
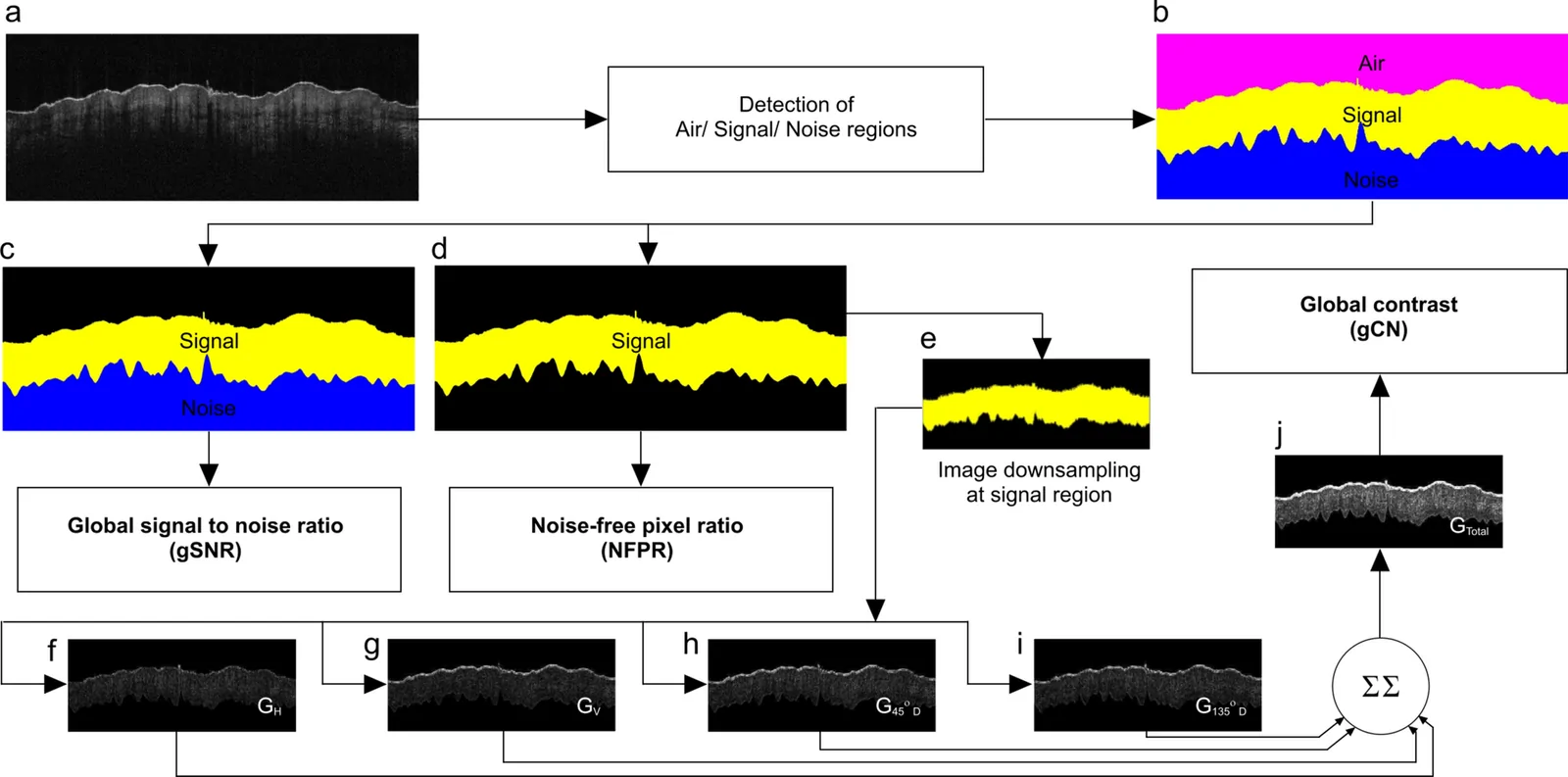
Flowchart of the proposed quality metrics calculations. (**a**) Original OCT skin image, (**b**) image partitioned into three regions of air, signal, and noise, (**c**) signal and noise region images used to calculate gSNR, (**d**) signal region image used to calculate NFPR, (**e**) down-sampled version of the signal region image. By calculating the absolute intensity difference of each pixel from the down-sampled signal image with its four neighboring pixels in four directions, four gradient images can be generated: (**f**) horizontal gradient map, (**g**) vertical gradient map, (**h**) 45° diagonal gradient map, and (**i**) 135° diagonal gradient map. For visualization purposes, gradient values are rescaled to enhance contrast and highlight spatial variations of three regions is the outcome. If the first detected region has the lowest mean CV value, it is classified as signal (Part 2), and the subsequent regions are classified as noise regions (see Figure).

The algorithm suggested for OCT image segmentation (described in Fig. 2), with input and output samples displayed in Fig. 3a and b, enables the creation of novel quality metrics for assessing OCT images in a manner that is not dependent on location.

#### Noise-free pixel ratio

The noise-free pixel ratio (NFPR) is defined as the ratio of the number of pixels in the signal region to the number of pixels in the entire image*.*

#### Global SNR (gSNR)

The regions of signal and noise depicted in Fig. 3c serve as the basis for establishing the metric defined as global SNR (gSNR). gSNR is defined as a depth-aggregated measure of signal strength relative to the noise floor. Let *z* denote axial depth positions where sufficient signal pixels exist. For each depth z, signal pixel intensities are sorted, and the median of the top K intensities is selected as a robust estimate of signal strength, denoted as *I*_*med*_*(z)*. The global noise level is characterized by the standard deviation *σ*_*n*_ of all pixels classified as noise. The gSNR is then defined as:

1 $$\mathrm{gSNR}=10*log10\sum_{z\epsilon Z}\frac{{I}_{med}(z)}{{\sigma}_{n}},$$

#### Global contrast (gCN)

Contrast refers to the difference in brightness between different regions of the image. Here, we used the down-sampled version of the OCT image in which the intensity of all air and noise pixels has been assigned to zero.

This downsampled image is called the signal image. We used the built-in MATLAB function, ‘imresize’^37^ to obtain a downsampled version of the signal image (Fig. 3e). This resizing step was not performed by direct pixel decimation; rather, imresize was used with its default anti-aliasing behavior during image shrinking. The purpose of this step was to reduce high-frequency fluctuations, including speckle-related variations, and to capture coarse structural contrast at a larger spatial scale for the calculation of gCN. Starting at the upper left corner, in each pixel $\left(\mathrm{i},\mathrm{j}\right)$ of the resized image, we calculated the absolute intensity difference of that pixel with its four neighbor pixels in four forward directions: horizontal (i, j + 1), 45° diagonal (i + 1, j + 1), vertical (i + 1, j), and 135° diagonal (i + 1, j-1). Next, we sum these four values to determine the contrast for the (i, j) pixel, which we denote as *G(i,j)*_*Total*_. Figure 3f, g, h, and i show gradient maps in the four forward directions, and Fig. 3j shows the full gradient map. The gCN of the image is then calculated by averaging non-zero elements in *G(i,j)*_*Total*_ as shown in Eq. 2.

2 $$\mathrm{gCN}=\frac{\sum_{i=1}^{m}\sum_{j=1}^{n}{G(i,j)}_{Total}}{N^{\prime}},$$

where *m* and *n* are the dimensions of the resized image and *N'* is the number of non-zero elements of *G(i,j)*_*Total*_.

### Image quality alteration strategy

We used different datasets to evaluate our proposed image quality metrics: this enabled us to analyze image quality one aspect at a time. In order to evaluate NFPR and gSNR, we varied the laser power output on the OCT machine. Increased laser power increased penetration depth, increasing the number of pixels in the signal region (and the NFPR)^38^. Increased laser power is also known to correlate with increased SNR. We therefore acquired skin images at nine different laser powers (109 mW, 163 mW, 218 mW, 272 mW mW, 323 mW, 382 mW, 435 mW, 489 mW and 544 mW). To evaluate gCN, a different strategy is required. Initially, rigid registration based on normalized cross-correlation (NCC)^39,40^ was performed to correct for global motion, such as jitter or pulsation, between frames. Subsequently, 25 images from the same acquisition were averaged to generate a reference image with reduced stochastic variability, enabling controlled evaluation of contrast-related changes. This step was introduced solely to create a controlled dataset for evaluating the behavior of the proposed metrics under varying contrast conditions and does not reflect a requirement of the metrics themselves. Finally, the built-in MATLAB function, ‘adapthisteq’^41^, was applied to modify the contrast of the images using Contrast-Limited Adaptive Histogram Equalization (CLAHE), a well-known image processing technique for increasing contrast^42^. By adjusting the contrast factor of this algorithm from 0.001 to 0.018, 9 sets of OCT images with different levels of contrast were obtained. Finally, for the purpose of assessing the alignment of the new metrics with human visual perception, we employed 4 distinct filters, designated as Kuwahara, Wiener, Non-Local Means (NLM) and Block-matching and 3D Filtering (BM3D) that are capable of significantly diminishing speckle noise in images^2,43,44^ and produced a set of visually comparable denoised images of the same cross-section. By systematically applying these filters, and utilizing varying parameter values, we were able to produce images that exhibited variations in visually perceptible image quality.

## Results

**Fig. 4:**
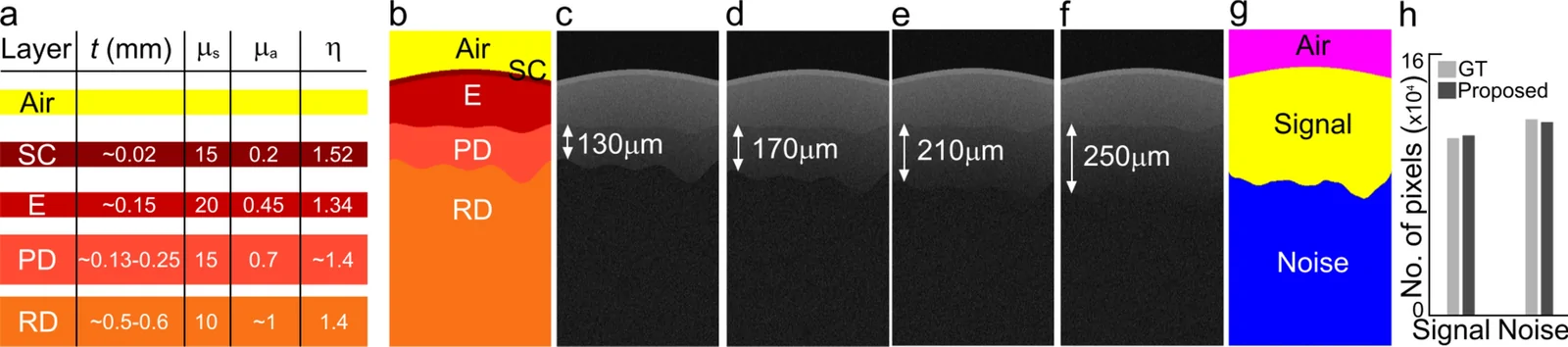
Validation of the segmentation algorithm using simulated OCT images. (**a**) Optical properties and thicknesses of skin layers—scattering coefficient (μs), absorption coefficient (μa), refractive index (n), and thickness (t)—were derived from published literature, (**b**) schematic representation of the layer organization: stratum corneum (SC), epidermis (E), papillary dermis (PD), and reticular dermis (RD), (**c**–**f**) simulated OCT images where the thickness of the PD layer was progressively increased to create a gradual loss of visual contrast between the PD-RD boundary, (**g**) segmentation output for the most challenging case (**f**), showing the algorithm’s performance under minimal boundary visibility, (**h**) comparison between true and estimated signal/noise pixel counts, supporting the quantitative evaluation of segmentation accuracy.

To rigorously assess the performance of the proposed quality metrics, it was first necessary to validate the accuracy of the segmentation algorithm. This required a test image with clearly defined boundaries between skin layers, mimicking the structural and optical properties of real OCT images, including the characteristic exponential signal decay with depth. To this end, we generated synthetic OCT skin images using Monte Carlo simulations, consisting of four distinct layers: SC, epidermis (E), papillary dermis (PD), and reticular dermis (RD)^45,46^. The inherent axial jitter and jagged behavior of the interface between PD and RD were recognized as fundamental consequences of the stochastic nature of MC simulations. It was understood that these axial fluctuations are driven by the local statistical realization of scattering events, where the perceived boundary is shifted superiorly due to the system’s point spread function (PSF) and coherent crosstalk, or inferiorly due to Poisson-governed local rarefactions of scatterers. To minimize these stochastic artifacts while preserving the physical integrity of the simulation, the number of launched photons per A-scan was significantly increased. This strategy was employed to facilitate the convergence of the backscattered signal toward its statistical expected value, thereby filling “stochastic voids” and ensuring that the high-variance regime of the RD initiated as accurately as possible at the physical transition. The optical parameters of each layer—scattering coefficient (μs), absorption coefficient (μa), refractive index (n), and thickness (t)—were derived from published literature (Fig. 4a), with layer organization illustrated in Fig. 4b^47^. To simulate a challenging boundary-detection scenario, we progressively increased the thickness of the PD layer to the point where the PD–RD boundary became visually indistinguishable (Fig. 4c–f).

This enabled evaluation of whether the segmentation algorithm could still correctly delineate this blurred interface. Figure 4f, representing the most challenging case, was used as the input to the segmentation algorithm, with the result shown in Fig. 4g. The algorithm demonstrated perfect accuracy in detecting the air–tissue interface. To further evaluate its ability to distinguish between signal-bearing regions (E, PD, RD) and noise-dominated areas (where OCT signal is no longer reliable), we compared the number of true signal and noise pixels with those estimated by the algorithm. This comparison, shown in Fig. 4h, is critical because the proposed quality metrics depend directly on accurate identification of these regions. The segmentation algorithm achieved an accuracy of 98.74% in distinguishing signal from noise region pixels in the simulated dataset, where ground-truth labels are available. This result demonstrates that the proposed segmentation approach performs effectively under controlled conditions. However, this accuracy reflects performance on simulated data and may not directly translate to real OCT images, where the transition between signal and noise is inherently gradual and ambiguous. In practical OCT data, segmentation should therefore be interpreted as an approximate statistical separation rather than a precise ground-truth classification.

**Fig. 5:**
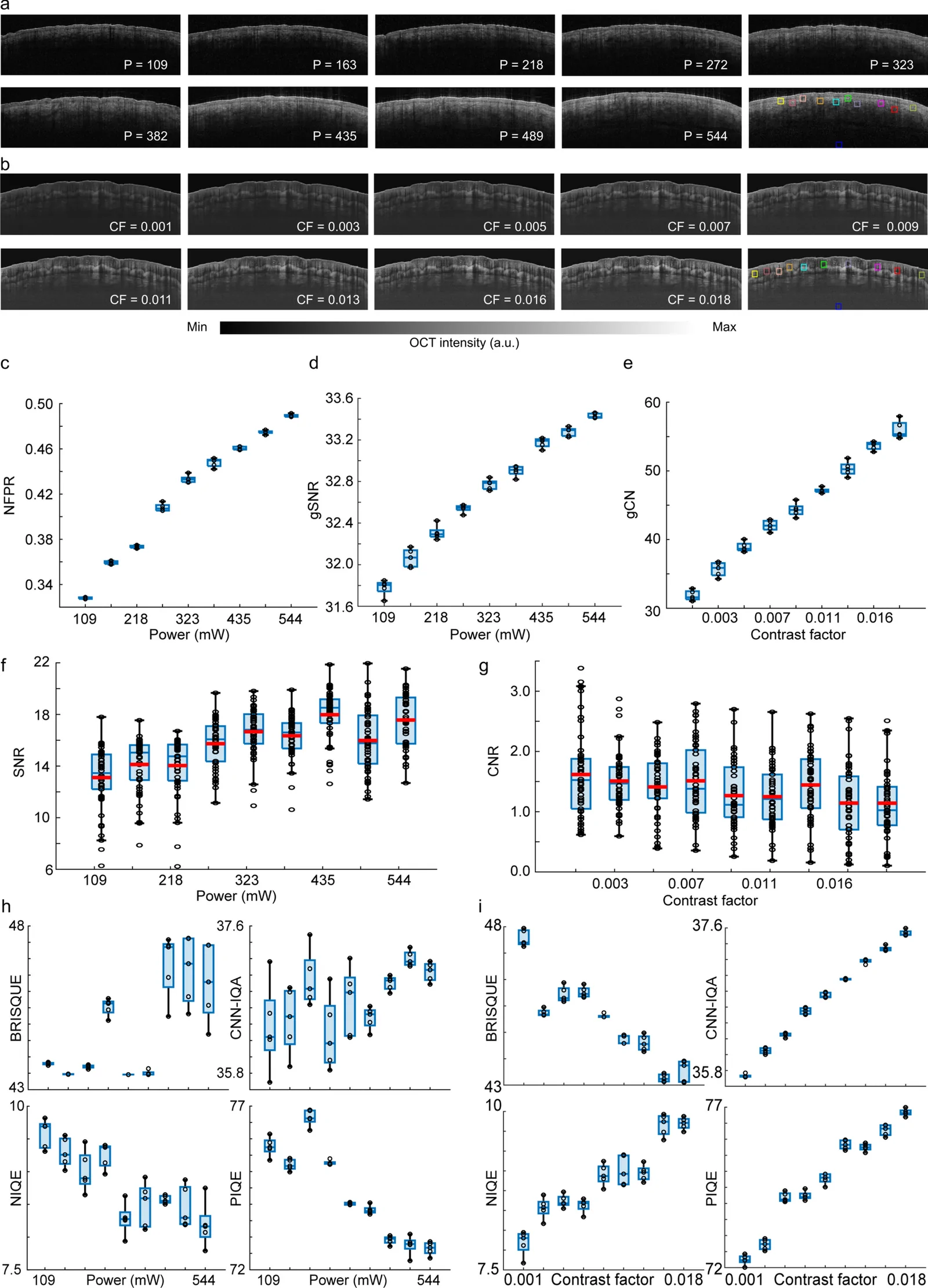
OCT images obtained by: (**a**) imaging the same section using an imaging system with various laser powers (values of P are in mW), (**b**) applying Contrast-Limited Adaptive Histogram Equalization (CLAHE) with different contrast factors. P and CF represent laser power and contrast factor, respectively. The final image in each set illustrates the representative locations of signal and noise regions used for conventional ROI-based metric calculations. The results of calculating the quality metrics for the OCT images are shown as box plots: the (**c**) NFPR, (**d**) gSNR, (**e**) gCN, (**f**) SNR, and (**g**) CNR. For SNR and CNR, both the median (blue line) and mean (red line) are depicted. Also, the results of calculating No-reference Image Quality Assessment (NR-IQA) for the OCT images with different (**h**) laser powers, and (**i**) contrast factors are shown as box plots.

Next, to assess the newly introduced metrics, a range of OCT images with varying quality was analyzed. For NFPR and gSNR, a subset of five images from each of the nine sets of OCT images collected at different power levels was selected to provide a representative evaluation across conditions (an image from each set is shown in Fig. 5a). To evaluate gCN, a second dataset consisting of multiple image collections with varying contrast levels was used, where contrast was modified using CLAHE. Five sets of images with different contrast levels were selected from this dataset (one set is shown in Fig. 5b). This selection was intended to provide a controlled proof-of-concept evaluation of metric behavior under systematically varied imaging conditions, rather than to establish population-level statistical conclusions.

The relationship between the rise in laser power and the improvement in imaging depth is clearly demonstrated in Fig. 5a. It was anticipated that the NFPR metric would demonstrate this fact quantitatively. Figure 5c demonstrates that NFPR increases with increasing laser power, indicating the effectiveness of this metric. Next, within this collection of images, the gSNR evaluation was performed using Eq. 1, and the corresponding gSNR values are depicted in Fig. 5d. The rise in gSNR values correlates very well with the increase in laser power. The gCN evaluation was performed using the second dataset of images (Fig. 5b). Equation 2 was used to calculate gCN, and the outcomes are illustrated in Fig. 5e.

As can be seen in Fig. 5b, increasing the contrast factor of the CLAHE algorithm does correspond to an increase in the level of contrast (see Fig. 5b). This is clearly visible in the trend obtained for gCN values (Fig. 5e), and as a result, the efficacy of this metric is also proven.

Subsequently, in order to make a comparison between the new metrics and conventional metrics, SNR and CNR were also determined. To illustrate the variability of SNR, we found values for SNR for each of ten 41 × 41 pixel regions in each of the five images used for calculating gSNR. The final image in Fig. 5a displays the positions of the ten signal regions, which are depicted in various colors, and the noise region, from a selected image. For each laser power, the SNR values were calculated for each region in each image and depicted as separate box plots with the mean values denoted by red lines (Fig. 5f). To calculate CNR, the procedure was repeated in each of the images that were used to calculate gCN. Again, an equal number of regions (from regions which appeared visually to represent the signal region) with matching dimensions were selected in the images and mean and variance and CNR values were obtained. The regions are represented by different colors in the last image of Fig. 5b. Figure 5g displays separate box plots with the mean values indicated by red lines for each contrast level, illustrating the calculated CNR values for all the selected regions in the images. Both Fig. 5f and g show the results of analysis of 50 different regions from five images at each power level (for SNR) and contrast level (for CNR).

The proposed metrics exhibit more consistent behavior across different images and acquisition conditions compared to conventional ROI-based metrics. In particular, gSNR demonstrates a clear and monotonic relationship with increasing laser power, consistent with expected improvements in signal penetration. In contrast, SNR values show greater variability across different ROI selections, reflecting their sensitivity to spatial sampling. By contrast, the conventional SNR values show greater variability across ROI selections and a less consistent relationship with laser power. Additionally, as can be seen in the introduction (Fig. 1), there exists wide variation in the SNR values among selected regions within a single image. In contrast, for a given laser power level, there is minimal variation of gSNR values even between different images. These observations suggest that gSNR provides a more stable and consistent representation of signal changes under the tested conditions. Next, the gCN metric exhibits a more consistent relationship with increasing contrast levels than the conventional CNR metric under the tested conditions^42^. gCN ignores noise entirely, focusing on a global averaging of the contrast between small, nearby parcels in the signal region. By contrast, CNR displays the difference in mean intensity divided by the sum of the variance of both the signal and background regions. While difference in mean intensity will increase in images enhanced by CLAHE, the variance will increase more, leading to the downward trend shown in Fig. 5g. The gCN values exhibit a consistent upward trend with increasing contrast levels, whereas the CNR values show less consistent behavior across ROI selections. Furthermore, there is a substantial degree of variability in the CNR values for every contrast level, leading to a lack of clear differentiation between the various contrast levels. Moreover, the CNR values do not show a consistent monotonic relationship with the imposed contrast changes, which limits their interpretability in this experimental setting. By contrast, there is minimal variation of gCN values between different images obtained at each contrast level, indicating the increased robustness of this metric compared to CNR.

The results for between the proposed metrics and the mentioned NR-IQA metrics, four standard benchmarks, NIQE, BRISQUE, PIQE, and CNN-IQA, were also analyzed:. First, these metrics were applied to the dataset shown in Fig. 5a, where the laser power was varied, and the results are presented in Fig. 5h, demonstrating that these metrics failed to accurately assess image quality under different optical power levels. This poor performance can be attributed to the fact that these tools were primarily trained on natural scene statistics rather than medical imaging. Consequently, due to internal normalization processes, variations in overall light levels are often ignored, and the specific speckle noise of OCT is frequently misinterpreted as structural detail. Ultimately, these algorithms prioritize aesthetic “beauty” over the scientific accuracy required for medical diagnosis. Second, the performance of these benchmarks on the dataset in Fig. 5b, featuring varying contrast factors, is illustrated in Fig. 5i. Among these methods, only CNN-IQA was found to provide a reliable assessment, as its neural network architecture is capable of recognizing enhanced textures and sharpness as indicators of higher quality, and NIQE, BRISQUE, and PIQE were unable to provide accurate results; they perceive high contrast as an “unnatural” distortion, thus interpreting improved clarity as a loss of quality.

Finally, we sought to bridge the gap between objective image quality metrics and the subjective experience of viewing images with the expectation that reliable metrics would provide insights that could inform the development of more effective imaging technologies and standards. To achieve this objective, we employed a dataset generated from a variety of filters configured with distinct parameters that exhibited varying levels of SNR and contrast. Increased visual perception generally correlates with higher SNR and contrast. Higher SNR values typically indicate clearer images, while lower values suggest that noise may obscure important details. In addition to SNR, contrast plays a vital role in visual perception. It refers to the difference in luminance or color that makes an object distinguishable from other objects and the background. Images with high contrast are generally perceived as more vibrant and detailed, while those with low contrast may appear flat and lacking in depth.

**Fig. 6:**
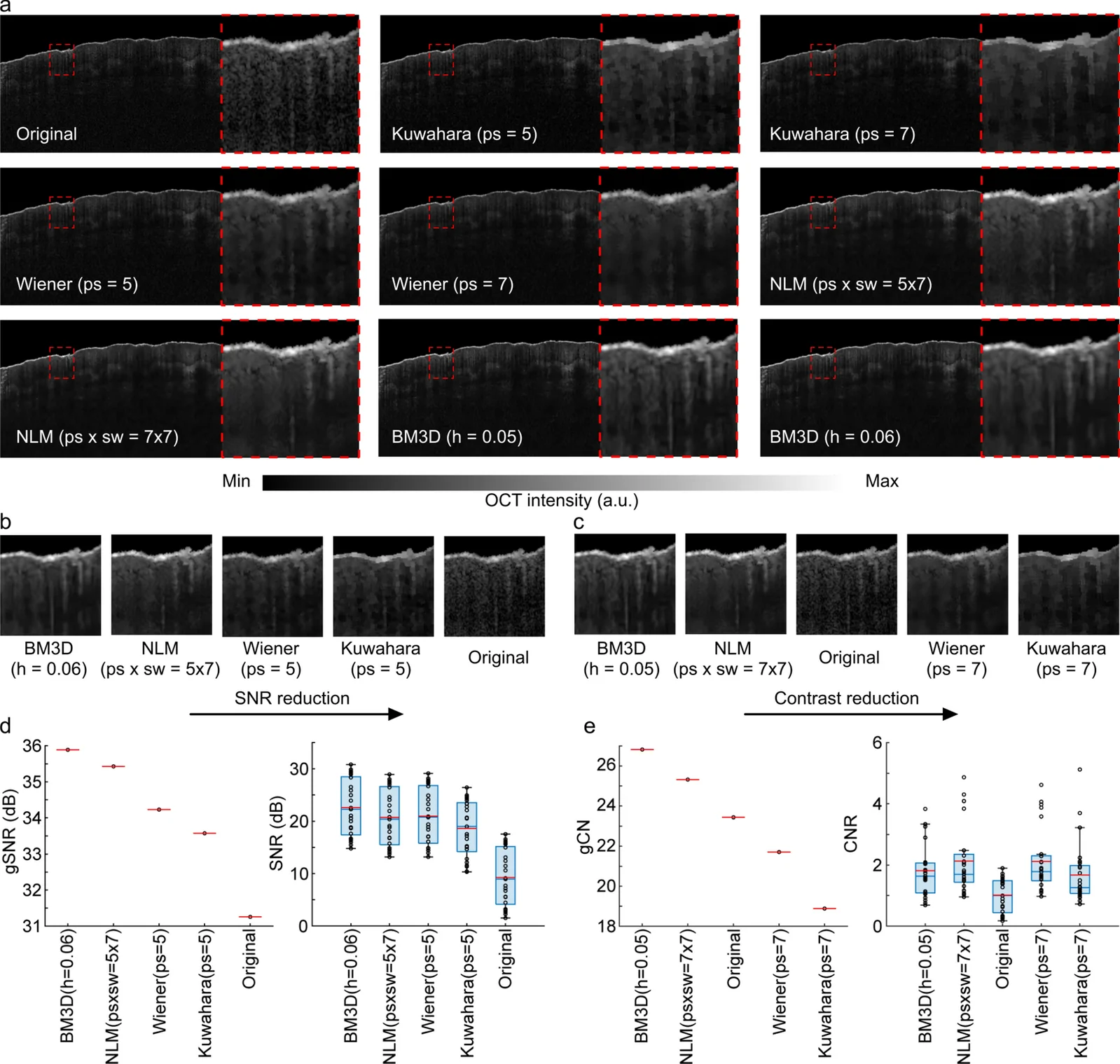
The comparison of traditional and proposed image quality metrics and human visual perception. (**a**) OCT images obtained by applying denoising filters including: Kuwahara, Wiener, Non-Local Means (NLM) and Block-matching and 3D Filtering (BM3D), utilizing two distinct parameter values for each filter, (**b**) inserts of the denoised images and the original image arranged in descending order based on the extent of visual disparity between them, evaluated through (b) SNR and (**c**) contrast. Ps, sw and h represent patch size, search window and filter strength, respectively. The results of calculating the corresponding quality metrics for the OCT images shown as box plots: the (**d**) gSNR and SNR, (**e**) gCN and CNR. For SNR and CNR, both median (blue line) and mean (red line) are depicted.

To demonstrate the relationship between human visual perception, specifically regarding SNR and contrast, and the newly developed metrics, we categorized the two factors, SNR and contrast, into distinct groups. For each category, we chose one image enhanced by each of the four filters, ensuring that the selected images exhibited the most significant visual differentiation relative to the other images concerning the respective factors. For the Kuwahara and Wiener filters, we selected images with window size parameters of 5 and 7. In the case of the NLM filter, we utilized images where the search window parameter was set to 7, while the patch size parameters were 5 and 7. Finally, for the BM3D filter, we opted for images with h parameter values of 0.05 and 0.06, which determine the filter’s strength. This methodology led to the creation of 2 images for each filter, resulting in a total of 8 images which are presented alongside a magnified section of each in Fig. 6a.

We arranged these 8 images in order to increase visual perception, which also incidentally arrayed them from highest to lowest SNR. The outcomes of this configuration are illustrated in Fig. 6b and c, respectively, which includes enlarged sections of the images to enhance comprehension. In the initial group associated with SNR, both the common SNR and gSNR metrics were computed. These metrics correspond to images organized in a descending order of SNR, as illustrated in Fig. 6d. In the subsequent group, which pertains to contrast, the CNR and gCN were determined. The results, which relate to images arranged by decreasing contrast, are presented in Fig. 6e. Here, the calculation of the SNR and CNR was conducted using 25 regions, each measuring 15 × 15 pixels, designated as signal areas. Additionally, a single region of the same dimensions located in the lower section of the image was utilized to represent noise.

The new metrics align more closely with the human observer’s perception of image quality compared to the traditional metrics. The visual arrangement of the images, organized from the highest to the lowest SNR, reveals a consistent downward trend in gSNR, suggesting a strong relationship between this metric and human assessment of image quality. Furthermore, the visual distinctions among the images correlate well with the variations in gSNR values. In contrast, the trend observed in SNR is contrary to what one might intuitively expect. In addition, the SNR values across various regions of filtered images exhibit a high degree of similarity, making it challenging to identify the most effective filter. Moreover, in gCN, a clear and consistent decline is observable. The images, organized from highest to lowest perceived contrast, suggest that gCN correlates effectively with human assessments of image quality. The significant variation in the gCN values, which aligns with the visibly distinct differences among the images, further supports this notion. Conversely, as indicated by prior findings, CNR is not an appropriate metric for assessing the contrast, and the alterations in its values do not exhibit a decreasing pattern. The findings indicate that the previously established metrics, as illustrated in the introduction (Fig. 1), exhibit significant variations in their values across the selected regions within the same image. Furthermore, these values do not correspond well with human visual perception. In contrast, the newly proposed metrics, which provide a singular value for an image, demonstrate a strong alignment with human visual perception.

## Discussion

This study introduces a principled framework for OCT image quality assessment by redefining quality metrics in a statistically and physically grounded manner. Unlike conventional metrics such as SNR and CNR, which rely on arbitrarily selected regions of interest and are therefore sensitive to user bias and spatial sampling, the proposed global metrics—NFPR, gSNR, and gCN—are derived from a segmentation-driven representation of the entire OCT image. By explicitly partitioning the image into air, signal, and noise regions based on known OCT signal statistics, the framework enables quality assessment that reflects intrinsic image formation properties rather than localized intensity comparisons. Importantly, the proposed metrics are designed to capture distinct and complementary aspects of OCT image quality. NFPR reflects the proportion of pixels containing meaningful backscattered signal and can be interpreted as a surrogate for effective imaging depth and information content. gSNR quantifies signal strength relative to the global noise floor using robust statistics derived from the segmented signal region, while gCN captures structural contrast through spatial gradient statistics that are independent of absolute intensity scaling. These formulations are grounded in the statistical behavior of OCT signals, including the transition from structured signal regions to noise-dominated regions, making the metrics both interpretable and physically meaningful^48,49^.

We also note that the proposed framework differs conceptually from general-purpose NR-IQA methods, including BRISQUE, CNN-IQA, NIQE, and PIQE. While these NR-IQA approaches estimate perceptual image quality or distortion levels using natural-image statistical priors or learned image-quality models, they are not explicitly designed around the physics of OCT signal formation. Therefore, their output may not be directly interpretable in terms of OCT-specific factors such as depth-dependent signal attenuation, speckle behavior, tissue penetration, or the transition from signal-dominant to noise-dominant regions. In contrast, our framework leverages domain-specific OCT signal and noise characteristics to define metrics that are directly linked to acquisition conditions and image-formation behavior. As such, the proposed metrics are intended to complement NR-IQA methods by providing OCT-specific, interpretable measures for system evaluation and algorithm benchmarking^27,50,51^. These metrics are also intended to enhance the diagnostic performance of the developed algorithms^52–59^.

Our experimental design revealed several compelling findings. First, the strong linear correlations (R^2^ > 0.97) between the proposed metrics and physical image parameters (e.g., laser power or contrast level) underscore their sensitivity and robustness in quantifying image quality. Second, when tested against conventional metrics (SNR and CNR), our metrics demonstrated strong and consistent correlation with visually apparent changes in image quality while also avoiding the variability induced by subjective ROI selection, a key weakness of existing approaches. Third, we provided visual and statistical evidence that the new metrics consistently reflected quality changes induced by both acquisition settings and post-processing enhancements, showing high utility across a broad range of scenarios. It should be noted that conventional SNR and CNR metrics remain valid within carefully selected and well-defined regions of interest; however, their dependence on ROI selection introduces variability that the proposed global framework seeks to reduce. Importantly, the comparison with conventional metrics is not intended as a direct comparison of absolute numerical values, but rather as an assessment of relative trend consistency, variability, and sensitivity to controlled changes in acquisition and post-processing conditions. It should also be noted that the experimental evaluation was performed on a limited subset of images per condition, and therefore the observed trends should be interpreted as indicative rather than statistically exhaustive. In addition, the selection of images from each condition may introduce sampling bias, and future studies should incorporate larger datasets and more systematic sampling strategies to further validate the robustness of these observations.

An especially notable outcome is that these metrics can facilitate more objective benchmarking of novel image enhancement algorithms. As the biomedical imaging community increasingly incorporates AI-based denoising and super-resolution models^7–9,60–63^, having a robust, ROI-independent metric system is crucial. Our framework provides an immediate, interpretable, and quantifiable feedback mechanism that could accelerate algorithm development, parameter tuning, and clinical translation of image enhancement tools. Beyond image processing validation, the proposed metrics may have potential applications in several domains. In clinical workflows, they could support automated quality control by identifying suboptimal OCT scans during acquisition, thereby reducing the need for repeated imaging and improving efficiency. In longitudinal studies and multi-center datasets, global and standardized metrics such as those proposed here may help improve consistency across operators and imaging sessions.

Despite the promising performance of the proposed framework, several limitations should be acknowledged. First, the current study was conducted exclusively on OCT skin images, and therefore the experimental validation is restricted to this application. Although the proposed approach is motivated by general OCT signal formation principles, including depth-dependent attenuation and the statistical separation of signal- and noise-dominated regions, its performance must be rigorously evaluated on OCT images of other organs, such as the retina and coronary arteries, where tissue organization, boundary characteristics, and signal behavior may differ substantially. In particular, the use of coefficient of variation as a distinguishing feature between signal and noise regions relies on approximate statistical assumptions that may vary across imaging systems and acquisition settings, and should therefore be interpreted as a robust heuristic rather than a strict criterion. In addition, although the change-point locations are estimated automatically from each A-line, the maximum number of allowed change points was selected based on the expected statistical structure of skin OCT A-lines and may require adjustment or adaptive selection for tissue types with more complex signal organization. Second, although the dataset includes subjects with diverse ages, genders, and Fitzpatrick skin types, the sensitivity of the proposed algorithm to these biological variables has not been explicitly quantified; future studies should systematically evaluate whether skin pigmentation, age-related structural changes, or gender-associated dermal variations influence boundary detection and metric behavior across diverse populations. Third, broader hardware dependence remains to be investigated, as system-specific factors such as wavelength, axial and lateral resolution, reconstruction pipeline, noise floor, detector dynamic range, and other hardware characteristics may affect both segmentation performance and metric consistency. Therefore, further validation across additional anatomical sites, larger and more diverse datasets, and a wider range of OCT devices and configurations is necessary before the framework can be considered broadly generalizable.

Looking forward, there are several directions for further development. First, real-time integration of the proposed metrics into OCT acquisition systems could provide immediate feedback during imaging, improving quality assurance at the point of care. Second, coupling these metrics with machine learning models could enable automatic scan rejection, quality prediction, or even adaptive acquisition strategies. Third, the metrics can be adapted for volumetric OCT analysis, supporting 3D quality assessment for more comprehensive evaluations. Lastly, exploring the integration of these metrics into regulatory workflows or image repositories could standardize quality benchmarking across the OCT imaging ecosystem.

## Conclusion

This study introduces a paradigm shift in the evaluation of OCT image quality by addressing one of the field’s longstanding limitations: the dependence of conventional metrics on arbitrarily selected regions of interest. We developed a fully automated framework that segments OCT skin images into air, signal, and noise regions, enabling the calculation of three global, objective metrics-NFPR, gSNR, and gCN. These metrics offer reproducible, user-independent evaluations that correlate strongly with both physical acquisition parameters and human visual perception. Our results demonstrate that the proposed metrics significantly outperform traditional quality indicators in both sensitivity and consistency. By capturing statistical properties across the entire image, these metrics resolve the ambiguity and variability that have historically hindered the development and benchmarking of image processing algorithms in OCT. Importantly, the method is fast, fully automated, and adaptable, making it a practical and powerful tool for a wide range of clinical and research applications. The implications of this work are far-reaching. Beyond enabling objective comparisons of image enhancement techniques, the proposed framework lays the foundation for real-time image quality feedback during acquisition, automated scan rejection systems, and robust cross-platform image harmonization in multicenter studies. With minor adjustments, the algorithm is extendable to other OCT domains-including ophthalmology, cardiovascular imaging, and oncology-broadening its utility and impact.

## Data Availability

The datasets generated and/or analysed during the current study are not publicly available due to participant privacy and institutional restrictions, but are available from the corresponding author on reasonable request.
